# In Reply 'Multiple Episodes of Presyncope in a Pacemaker Dependent Patient: What is the Diagnosis?'

**Published:** 2010-07-20

**Authors:** Siva K Mulpuru, Cesare Saponieri

**Affiliations:** Division of Electrophysiology, Long Island College Hospital, Brooklyn, NY

**Keywords:** Presyncope, Pacemaker

We thank Dr Bharghava for careful reading of our article [1]. As highlighted by Dr Bhargava, Non Competitive Atrial Pacing (NCAP) algorithm is designed to prevent atrial arrhythmias by preventing atrial pacing within 300msec after a sensed atrial event in the post ventricular atrial refractory period (PVARP). The varying AV delays and fixed interval of 300msec between sensed atrial event (AR) in PVARP and subsequent paced atrial event (AP) nicely illustrate this algorithm. It is nominally turned on in all Medtronic dual chamber pacemakers as in our patient (Figure 1). Other manufactures call it by different names. In Biotronik devices, the algorithm is termed as Atrial Upper Rate and it delays A-Pace by 250msec after a sensed atrial event in functional refractory period.

We would like to point out few points from the tracing:

1. VA interval can be computed by subtracting AR-AS interval + paced AV delay from VV interval. For each beat the calculated VA interval is 290msec.
2. Despite changes in VV intervals, VA intervals are stable. Changes in VV predict subsequent changes in AA intervals suggestive of VA linking. Presence of VA linking argues against sinus rhythm (Figure 2).

On ventricular pacing threshold testing, distinct VA conduction was noted. We agree to the fact that complete anterograde AV block localized to the AV node is not associated with retrograde VA conduction. However up to 40% of the patients with complete anterograde AV block localized to the His-Purkinje system can have some degree of retrograde VA conduction as seen in our patient [2]. Turing NCAP off and reducing PVARP would lead to pacemaker mediated tachycardia at the upper tracking rate and may be increased incidence of atrial arrhythmias.

Documentation of retrograde VA conduction is essential for the diagnosis of RNRVAS. It is important to distinguish retrograde VA conduction with functional under sensing of sinus rhythm. We believe that they are two completely different problems with opposite solutions. Careful observations during ventricular pacing threshold testing may help distinguish the two conditions.

## Figures and Tables

**Figure 1:**
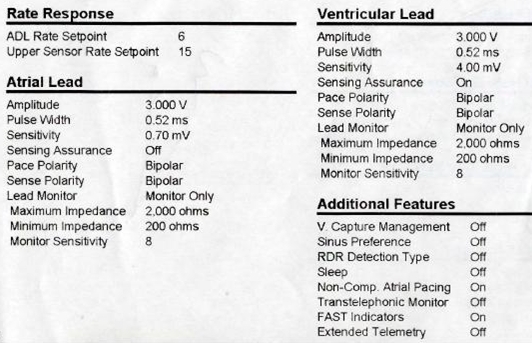
Non Competitive Atrial Pacing (NCAP) nominally turned on.

**Figure 2:**
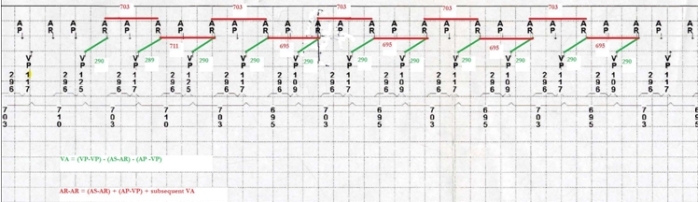
Tracing showing an episode of RNRVAS. VA intervals are marked in green and sensed inter atrial intervals are marked in red. Presence of VA linking along with dependence of A-A intervals on previous V-V intervals argue against under sensed sinus rhythm.
